# A pilot study using Tissue Velocity Ultrasound Imaging (TVI) to assess muscle activity pattern in patients with chronic trapezius myalgia

**DOI:** 10.1186/1471-2474-9-127

**Published:** 2008-09-24

**Authors:** Michael Peolsson, Britt Larsson, Lars-Åke Brodin, Björn Gerdle

**Affiliations:** 1 Department of Clinical and Experimental Medicine, Division of Rehabilitation Medicine, Faculty of Health Sciences, Linköping University, Linköping, Sweden; 2 School of Technology and Health, Royal Institute of Technology, Stockholm, Sweden; 3 Pain and rehabilitation Centre, University Hospital in Linköping, Linköping, Sweden

## Abstract

**Background:**

Different research techniques indicate alterations in muscle tissue and in neuromuscular control of aching muscles in patients with chronic localized pain. Ultrasound can be used for analysis of muscle tissue dynamics in clinical practice.

**Aim:**

This study introduces a new muscle tissue sensitive ultrasound technique in order to provide a new methodology for providing a description of local muscle changes. This method is applied to investigate trapezius muscle tissue response – especially with respect to specific regional deformation and deformation rates – during concentric shoulder elevation in patients with chronic trapezius myalgia and healthy controls before and after pain provocation.

**Methods:**

Patients with trapezius myalgia and healthy controls were analyzed using an ultrasound system equipped with tissue velocity imaging (TVI). The patients performed a standardized 3-cm concentric shoulder elevation before and after pain provocation/exercise at a standardized elevation tempo (30 bpm). A standardized region of interest (ROI), an ellipsis with a size that captures the upper and lower fascia of the trapezius muscle (4 cm width) at rest, was placed in the first frame of the loop registration of the elevation. The ROI was re-anchored frame by frame following the same anatomical landmark in the basal fascia during all frames of the concentric phase. In cardiac measurement, tissue velocities are measured in the axial projection towards and against the probe where red colour represents shortening and red lengthening. In the case of measuring the trapezius muscle, tissue deformation measurements are made orthogonally, thus, indirectly. Based on the assumption of muscle volume incompressibility, blue represents tissue contraction and red relaxation. Within the ROI, two variables were calculated as a function of time: *deformation *and *deformation rate*. Hereafter, max, mean, and quadratic mean values (RMS) of each variable were calculated and compared before and after pain provocation/exercise.

**Results:**

This new methodology seems valuable when looking at local muscle changes and studying the mechanism behind chronic muscle pain. The univariate analyses indicate that patients with chronic trapezius myalgia after pain provocation due to exercise at group level showed decreased strain and unchanged strain rate while healthy controls had unchanged strain and increased strain rate. However, the multivariate analysis indicates that most patients showed lower levels according to both strain and strain rate after exercise compared to most controls.

**Conclusion:**

Tissue velocity imaging can help describe musculoskeletal tissue activity and dynamics in patients with chronic pain conditions. An altered muscle tissue dynamic after pain provocation/exercise among the majority of trapezius myalgia patients compared with the healthy controls was found.

## Background

Musculoskeletal disorders comprise one of the most common and costly public health issues in western countries [1]. In the Nordic countries and Holland, the cost has been estimated to range from 0.5 to 2% of the GNP [2]. Among these disorders, persistent neck and shoulder complaints/pain are very common. The basis for the diagnostic criteria of neck and shoulder myalgia is relatively vague and the diagnostic terminology and methods for assessment of neck and upper limb musculoskeletal disorders vary. The patients' complaints and the manual clinical examination are the most important instruments in the process of diagnosis. When the criteria for assessing pain and non-articular soft-tissue disorders of neck and upper limb were reviewed, it was concluded that the diagnosis relies heavily on the clinical opinions of the investigators [3]. Accurate and standardised diagnosis of neck and shoulder myalgia is a prerequisite for adequate interventions and would reasonably advance the management of these disorders, but at present no such generally accepted and applied system of diagnosis exists.

Local muscular processes may explain chronic pain, but the role of these processes are questioned by some authors [4]. For example, in longstanding neck-shoulder pain the muscular component is difficult to confirm in clinical practice. Different studies indicate changes in the muscle tissue and/or disturbed neuro-muscular control in patients with chronic pain. Hence, ragged-red fibres (RR-fibres are muscle fibres with signs of a disturbed oxidative metabolism) and fibres that lack cyto-chrome-c-oxidase have been associated with myalgia [5-8]. Patients with chronic work-related trapezius myalgia and myofascial trapezius pain have changed interstitial milieus in the aching muscle [9-12]. The electrical activity that precedes a muscle contraction (electromyography (EMG)) can be used to investigate aspects of neuro-muscular control. Surface-EMG is in a complex way determined by both central factors and peripheral factors of the muscle fibres. In clinical management of patients with chronic pain, it is often implicit that painful and tender muscles have increased activity and as a consequence treatments are applied to reduce the supposed hyperactivity and hereby the pain. Increased EMG activity has been found during *dynamic *activity in parts of the contraction cycle [13-17]. Using multichannel surface EMG inhomogeneities in spatial changes in motor unit recruitment have been reported [18] and acute pain results in reorganization of activity pattern among trapezius muscle subdivisions [19].

Studies with different research techniques indicate alterations in muscle tissue and in neuromuscular control of the aching muscle in patients with chronic localized pain. Analysing muscle tissue movements during dynamic muscle performances might be of great value in clinical practise. Ultrasound can provide such an opportunity. Several methods have been developed to describe tissue deformation (strain) [20,21]. Elastography [22-24] is a technique introduced for this diagnostic purpose. In this case, mainly external and thus known forces are applied to measure elastic components in tissue. A limitation lies in that displacements and strain within the object should be limited. Improvements have been made during the late 1990s where methods have been developed within cardiology measuring as well as movements and deformation parameters [25-29]. This method has been tested for validity and reproducibility with respect to regional heart function [30]. Musculoskeletal contraction and relaxation has been described based on the length of muscle fascicle and angle of pennation [31-35].

A new type of software package for analyzing tissue functionality based on ultrasound has been developed within echocardiography [27,28]. This software package provides the opportunity to calculate segmental lengthening and/or shortening within the myocardium over time. Algorithms within this package calculate segmental movements as well as deformations and present the result in patterns of segmental coordination over the heart cycle. The results are also graphically and quantitatively described in terms of contraction and relaxation phases during the heart cycle. As a result, active and passive segments may be discerned and as a consequence functional parameters such as patterns of myocardial dysfunction may be identified. For example, as a consequence of cardiac infarction or ischemia, different patterns of dyskinesia may occur and consequently pharmacological treatment may be evaluated on the basis of alteration of heart dynamics. An early application study before the strain rate imaging (SRI) concept was introduced according to skeletal muscle tissue was performed by Grubb et al. [36]. They applied the tissue velocity imaging (TVI) technique in isolation was applied for isotonic and isometric contractions. They concluded that discrimination between active and a passive movement was possible.

## Aim

The overall aim of this pilot study was to introduce a new muscle tissue velocity sensitive ultrasound technique and to apply this technique to investigate trapezius muscle tissue response during concentric shoulder elevation in patients with chronic trapezius myalgia and healthy controls. In order to exemplify the technique regional deformation and deformation rates are considered.

## Methods

### Summary of Methods

This study introduces variables to describe tissue motion and tissue deformation based on ultrasound-generated images (GE-medical Vivid 7, equipped with Tissue velocity imaging (TVI)). The methodology was used on 14 female patients with trapezius myalgia and 13 healthy controls when performing a 3-cm concentric shoulder elevation before and after pain provocation/exercise. A standardized region of interest (ROI) had a shape of an ellipsis and with a size that captures the upper and lower fascia of the trapezius muscle (4-cm width) at rest. The ROI was placed in the first frame of the loop registering the elevation and was subsequently re-anchored frame by frame in all frames of the concentric phase according to a certain acoustic pattern in the basal fascia that was used as a reference tag. In this way, the same tissue area was followed during all frames of the concentric phase and the quantitative measurements were calculated within the same tissue area. Within the ROI, two variables were calculated as a function of time: *deformation *and *deformation rate*. Hereafter, max, mean, and quadratic mean values (RMS) of each variable were calculated and compared before and after pain provocation/exercise.

#### The ultrasound equipment

A GE-Vivid 7 was equipped with separate non-commercial research software. A 12 MHz linear multi-hertz probe was used for all registrations. All analyses were made post-processed on custom-made non-commercial software.

#### The placement of the probe

To optimize the reproducibility of location of the probe, the SENIAM standard point of the trapezius [37] was used to centre the probe. The line from C7 to the edge of acromion was measured. This distance was divided by two, and the highest point on the shoulder belly at this distance was marked. The linear probe, measuring 4-cm long and 1-cm wide, was put in a coronal position over the landmark. Furthermore, the contour of the probe was drawn at the first placement. The angle of the probe was placed as close to the coronal plane as possible. Care was taken to ensure the same projection image was on the screen in all images.

#### EMG equipment

In this study, a single bi-polar EMG electrode (Centre to centre distance: 17 mm; Ambu, Ballerud, Denmark) was used to synchronize the ultrasound registration. The EMG electrode was placed at the medial edge of the probe 2 cm from the SENIAM point. The EMG was used as a reference indicating the electrical activation of muscle tissue. It was also used to ensure that the trapezius muscle was relaxed before and after the shoulder activation.

#### Tissue velocity imaging

**Figure 1:**
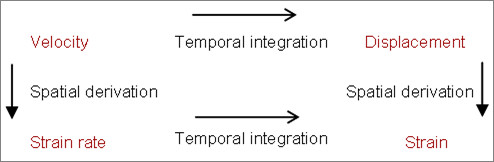
**The relationships between velocity, displacement, strain rate, and strain**. The figure illustrates the mathematical relationships between the four modalities that are used to describe velocity and deformation.

To describe tissue activity, two fundamental concepts are used: 'movement' and 'deformation'. The most important difference between them is whether there is a presence of acceleration. To use a metaphor, imagine a train with some cabins. If no accelerating/decelerating force is present, the train will travel with constant speed and at a certain time point a certain distance is reached. The parameters used to describe this situation by way of ultrasound loops is velocity and displacement. However, when the train alters its speed, starts or brakes, an acceleration or deceleration occurs and the distance between the cabins will either be elongated or compressed. Thus, a deformation between the cabins of the train set occurs. Deformation is measured according to rest values and labelled strain when analyzing the ultrasound images, and the rate of deformation is labelled strain rate. In summary, to describe movement the terms velocity and displacement are used; to describe deformation, the terms strain and strain rate are used. The relationships between the four variables are given in Figure 1.

Because the skeletal muscle is an elastic entity, functional movements are part of variations in the neuro-motor activation pattern. This will result in a fine-tuned muscle tissue response. This response can be described in terms of compression or elongation. When a tissue is compressed, shortened, a contraction is present. Consequently tissue elongation is related to relaxation.

#### Strain

The concept of strain was originally used by Mirsky and Parmley (1973) [38] to describe elasticity and stiffness in the heart muscle as an altered dimension between an object at rest and a force-induced condition calculated according to (strain) = $\frac{L-L_{0}}{L_{0}}$. *L*_0 _is the original length and *L *is the measured new length of the object. As a consequence, negative strain implicates compression and positive elongation.

#### Strain rate

**Figure 2:**
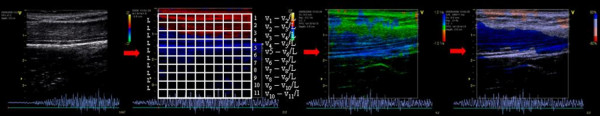
**The principle of calculations of the superimposed colour-coded images; see text for explanations**. The images above illustrate the calculating principle providing data to be presented in colour-coded images presenting regional strain rate and strain (see text for detailed explanations).

Strain rate [27,39] is the rate by which a deformation occurs, the change in strain as a function of time. A small alteration in length (dL) within a small time segment (dt) is related to the velocities of the endpoints of the object: $dL=\frac{v_{1}-v_{2}}{L}$. Using this technique in the ultrasound loops, the image lines are divided in small distances (offset) in this study by 8 mm and regional velocities within each offset is calculated several times. Hereafter the mean value of the calculations within each offset is calculated. This principle will be further described below. The velocities are measured in the axial direction of the ultrasound beam (Figure 2).

#### From grey scale image to strain rate and strain imaging

The graphical ultrasound interface presents both a qualitative visualization and quantitative results of the described variables. The qualitative information in gained from a colour-coded superimposed image calculated from the velocity calculations described above. Hence, by stacking all images as a function of time, the alteration due to muscle response can be visualized (Figure 2). The quantitative results stem from marking an area, a region of interest, (ROI) within the tissue in the initial frame of the loop; therefore, calculations are limited according to the ROI. As a result of the ROI being followed frame-by-frame, a quantitative curve according to each parameter is presented.

The calculation principle is illustrated in Figure 2. The color-coded images stem from measuring differences between transmitted and received signals along the image lines (vertical lines in the second image). The results from the received signals are converted to a specific colour representing the magnitude and direction of the velocities. To receive the strain rate image, the image lines in themselves are divided according to a chosen offset (sample length), a pattern that forms a grid. Regional velocities are measured every 0.5 mm and the average of the regional velocities within each cell is color coded. As a result, strain rate imaging (3^rd ^image in Figure 2) is a fine-tuned dynamic tissue mapping compared to the velocity images (2^nd ^image in Figure 2; the red/blue image behind the grid), which only visualizes global velocities towards and away from the probe. The fourth image in Figure 2 visualizes the deformation process. Note that the presented images in Figure 2 are extracted from the loop covering approximately 300 frames.

**Figure 3:**
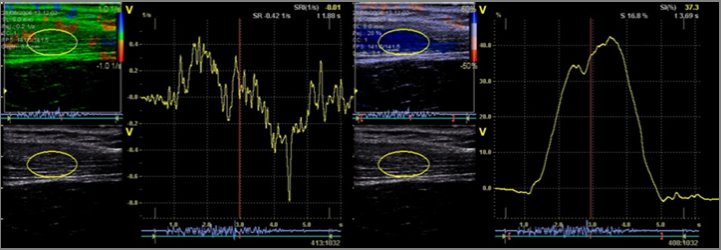
**Quantification according to strain rate and strain**. The figure above describes a multimodal presentation and includes qualitative and quantitative information calculated according to the yellow ROI. The left image denotes strain rate and the right image shows strain.

In the first image from the left, the grey scale image of the trapezius muscle is seen. The second image is the velocity mapping where the blue color represents velocities moving away from the probe and red towards the probe. To illustrate how the third image in Figure 3 is calculated, a grid has been placed in the velocity image. When calculating regional velocities within each cell of the grid, the colour-coded result is presented as a superimposed image in the third image. Here, a more fine-tuned activity pattern arises where yellow/green represents passive tissue segments and blue represents contractile segments. The red color marks relaxation and follows the same rules (not present in the example above). Note that the blue color changes in nuance in different parts of the image. Deeper blue segments have higher strain rate than lighter blue nuances. Hence, alterations in the difference in the degree of the speed of segmental tissue contraction is visualized (note also that these images are extracted from a loop covering the whole shoulder elevation). Consequently, the relaxation phase is color coded in a similar manner in different nuances of red, representing various degrees of tissue relaxation (not shown here). It is important to emphasize that this software is developed for measuring tissue velocities in the heart muscle. In this case, heart tissue velocities are measured in the axial direction, i.e., towards and away from the probe. In the original software package, the coding of tissue velocity direction is coloured red representing tissue velocities moving towards the probe (contraction) while blue represent tissue velocities moving away from the probe, i.e., tissue elongation. Hence, when measuring muscle tissue velocities orthogonally projected, velocities are always relative.

The fourth image in Figure 2 is the integrated strain rate curve, strain. The colour-coded image visualizes deep blue tissue segments exceeding 50% (by the analyzer possible choice between 5 – 50%) and medium blue segments deformed between 31 – 50%. Light blue indicates a more passive tissue segment.

Thus, the images in Figure 2 provide qualitative information about the contraction progress and thus the dynamics of tissue response within the trapezius muscle during a shoulder elevation. In turn, the strain rate modality illustrates both the recruitment order of very small tissue segments during the muscle contraction and the rate variation of the tissue responses during the elevation. Thus, strain rate could be seen as related to the neurological innervation of the muscle while strain can be seen as the functional tissue response where tissue activity pattern changes as a function of the progress and nature of the movement.

#### Quantification of tissue velocities from velocity images

To quantify the tissue activity, a region of interest (ROI) is manually placed in the first frame of the loop (in this case an ellipsis is chosen that captures the centre part of the trapezius). Average strain rate and strain values within the ROI are calculated frame-by-frame and presented as a function of time (Figure 3). As a result, a multimodal presentation is simultaneous presented: the original grey scale loop (lower left), the superimposed colour-coded loop (upper left), and the curve representing the average values of a ROI strain (right part).

#### Variables calculated from tissue velocity images in the present study

Five variables were calculated from the velocity, strain rate, and strain parameters. According to velocity and strain rate, the mean value and quadratic mean value (root mean square (RMS)) were calculated while mean, RMS, and maximum values consistent with strain were considered.

RMS is a statistical calculation that measures the magnitude of a varying curve. It is especially useful when the measurements vary between positive and negative. The RMS calculation for a collection of *n *values *x*_1_, *x*_2_, ..., *x*_*n *_is calculated using

$$x_{\text{rms}}\approx \sqrt{\frac{1}{n}\sum_{i=1}^{n}x_{i}^{2}}=\sqrt{\frac{x_{1}^{2}+x_{2}^{2}+\cdots +x_{n}^{2}}{n}}.$$

Furthermore, with respect to each variable the differences between the values before and after pain provocation were calculated. These variables form the basis of describing the results of the study.

### Subjects

To study the above described method, trapezius myalgia patients were recruited along with healthy controls. The patients were recruited among female out-patients who had been referred to the Pain and Rehabilitation Centre, University Hospital, Linköping, Sweden due to chronic neck and shoulder pain. Exclusion criteria were widespread pain, prior neck trauma, rheumatoid arthritis, drug or alcohol abuse, overweight, and grave depression. Medical records were assessed and former patients were identified as possible participants. These potential subjects were invited by mail to participate in the study. The participants were examined using a standardized clinical examination [40]. To ensure the myalgic trapezius muscle was the problem, the following inclusion criteria were used:

1. Pain present in the neck and shoulder region at the clinical examination two weeks before and on the experimental day;

2. A tight trapezius muscle: a feeling of stiffness in the descending region of the trapezius muscle reported by the patients during lateral flexion of the head;

3. Palpatory tenderness of the trapezius muscle of the painful side; and

4. Normal or slightly decreased range of movement of the cervical columna.

In all, 14 women – mean age: 38 years, median 40 years (range: 24–48); mean height: 168 cm, median height 168 cm (range: 158–175); mean weight: 65 kg, median weight 63 kg (range: 51–81) – comprised the pain group. No detailed information was available concerning the duration of chronic pain, but the medical records clearly reported ongoing pain of more than 6 months duration.

Thirteen healthy women without neck and shoulder pain in the same age groups – mean age: 43 years, median 41 years (range: 36–55); mean height 168 cm, median height 168 cm (range: 158–175 cm); mean weight: 65 kg, median weight 56 kg (range: 47–75) – were recruited among staff and students at the Linköping University Hospital. The healthy controls were investigated using the same exclusion criteria and clinical examination as the patients. One patient and one healthy control were excluded due to a shadowing scapula during the shoulder elevation according to the ultrasound registrations. One healthy control was excluded for not being present at the time of the tests.

The person handling the ultrasound equipment knew whether the subjects were patients or controls.

All participants of both groups gave their informed written consent and the study conformed to The Declaration of Helsinki, and the study was approved by the Ethical Committee of Linköping University (Dnr M103-06).

### Examination protocol

Two weeks after the clinical examination, the ultrasound (US) investigation was performed. The participants were asked not to use any medications due to pain 48 hours before the experimental day and were instructed not to perform any shoulder or neck-training exercise for 48 hours before the study, except for ordinary daily working and/or leisure duties. Furthermore, the participants were asked not to use nicotine or caffeine in any forms after midnight the day before the US examination.

#### Muscle contractions before pain provocation

The dynamic movement registered by the ultrasound image sequence (approximately 3-sec duration) covered the whole shoulder movement; i.e., both the concentric and the eccentric phase. The results of the present study are based on the concentric phase. The frame rate of the registrations is 141 frames per second giving a time resolution of approximately 7 ms. Thus, the rate at which the shoulder movement is registered is reasonably high enough to capture small intramuscular changes.

The subjects were asked to stand in an upright position with their arms hanging beside their body and their hands in a neutral position. The test subjects held a 1-kg dumbbell in the hand on the same side as the shoulder with pain; for the non-pain controls, the right arm was used. To standardize the shoulder elevation, a stand with an orthogonally placed level arm was used. The level arm was individually placed at each subject's shoulder at rest and a stop level was placed 3 cm above the shoulder in the elevation direction. The tempo of the movement was measured by a metronome and was set to 30 beats per minute. The subjects were instructed about the movements by the test leader and verbally led through the exercises concerning the tempo when performing the movements. Each subject performed two separate shoulder elevations at the start of the test. During the first elevation, the probe was held in a coronal position; during the second elevation, the probe was in a sagital position. Just a very short rest was present between the two elevations coinciding with the same procedure of the US loop. In this study, the coronal projection was used as this projection made it possible to follow a region of interest in the image sequence during the elevation. Thus, the before pain provocation procedure consisted of two subsequent elevations, which was registered by ultrasound. Hereafter, in mean three additional subsequent elevations were performed to achieve a pain intensity of 6 out of 10 according to a visual analogue scale (VAS; with the endpoints 0 = no pain and 10 = maximal pain intensity) without ultrasound registrations. When the patient had reached VAS 6, two subsequent ultrasound registrations were performed where ultrasound registrations were captured (i.e., after pain provocation procedure). As for the healthy controls, the procedure was 2 + 6 + 2 elevations where ultrasound registrations were performed during the first and last two elevations.

#### The pain/exercise provocation

The next step was pain/exercise provocation. The patients were asked to perform repetitive arm abductions holding the 1-kg dumbbell until the pain intensity according to VAS reached the level of 6. When the level was reached, the applicator provided two additional shoulder elevations in the same manner as at the start. First the applicator held the probe in a coronal position and then in the sagital projection. The number of additional repetitions varied slightly (between 2 and 3). As the healthy controls didn't report any pain at all, a standard choice of six repetitions was carried out by the controls. This choice was to make sure that the healthy subjects did not perform fewer repetitions than the patients (as the healthy patients were scheduled according to their possibility to attend in the study) without becoming fatigued.

#### Muscle contractions after pain/exercise provocation

Immediately after this provocation, the same procedure concerning shoulder elevation and probe position was repeated as before pain provocation. All US registrations were stored for post-process analysis.

### Statistics

All statistical evaluations were made using the statistical packages SPSS (version 12.0) for traditional statistics and SIMCA-P+ (version 11.5) for multivariate statistics. Generally, results in the text and tables are given as mean values ± one standard deviation (± 1SD). As the number of subjects incorporated in this pilot study was small, non-parametric statistics were used. Mann Whitney, Wilcoxon, and Fisher's Exact tests were used to test group differences and differences within groups.

Principal component analysis (PCA) was used for the multivariate analyses. PCA can be viewed as a multivariate correlation analysis. The PCA also gives the opportunity to multivariately investigate how subjects cluster into subgroups and how certain subgroups differ. PCA detects whether a number of variables reflect a smaller number of underlying components by linear combinations. A cross-validation method, which keeps part of the data out of the model development, is used to assess the predictive power of the model. The result of this procedure is a test of the significance of the components. A graphical plot is provided by the components. This plot could be seen of as a window (a plane) that is built on two t-vectors. All original variables are then projected onto this plane and receive a t-score represented by the coordinate values in plane. Next the orientation of the plane is calculated in relation to the original variable space where the angles between the plane and original space reveal the closeness between the model and the original space. Hence each variable receives a weight that expresses the impact each variable has on the model. Therefore, this projection window is called a *loading plot*. These projection techniques generate two corresponding plots, one for the observations and one for the variables. These plots are correlated according to the positions in one plot and correspond to the same position in the other plot. The *score plot *is used in the interpretation in such a way that correlation patterns among the observations are revealed, and the loading plot reveals the impact of each variable on the model. Variables that have high loadings (with either positive or negative sign) on the same component are inter-correlated. A component consists of a vector of numerical values between -1 and 1, referred to as loadings. When obtaining more than one component, the vectors are orthogonally projected to each other and thus uncorrelated. Variables that have high loadings (positive or negative sign) on the same component are inter-correlated. Items with high loadings (ignoring the sign) are considered to be of large or moderate importance for the component under consideration.

Two concepts are further used to describe the results: R^2 ^and Q^2^. R^2 ^describes the *goodness of fit *– the fraction of sum of squares of all the variables explained by a principal component. Q^2 ^describes the *goodness of prediction *– the fraction of the total variation of the variables that can be predicted by a principal component using cross validation methods. Outliers were identified using the two powerful methods available in SIMCA-P: score plots in combination with Hotelling's T^2 ^(identifies strong outliers) and distance to model in X-space (DModX) (identifies moderate outliers). In all statistical analysis, p ≤ 0.05 was regarded as significant.

## Results

### Group comparisons with respect to pain intensity

The pain intensity according to a 10-unit VAS scale was on average 2.5 (SD 0.94) for the TM group and 0 (SD 0) for the healthy control group before pain provocation. After pain provocation, the patient group scored 6.5 (SD 0.65), while the control group remained at VAS 0 (SD 0).

### Univariate analyses of ultrasound variables – group comparisons

Initially, a univariate analysis was performed (Table 1). No significant differences were found between patients and controls.

**Table 1:** Univariate analysis of the measured variables.

	**All (N = 24)**		**Controls**		**TM**		**Statistics**
**Group**			**(N = 11)**		**(N = 13)**		
**Variables**	**Mean**	**Std**	**Mean**	**Std**	**Mean**	**Std**	**(p-values)**
**Velocity (cm/s)**							
Velocity Mean bpp	-0.08	0.06	-0.06	0.06	-0.09	0.06	0.361
Velocity Mean app	-0.08	0.08	-0.09	0.11	-0.08	0.05	0.569
Velocity diff mean	0.00	0.02	0.03	0.05	0.01	0.01	0.459
							
Velocity RMS bpp	0.13	0.07	0.11	0.06	0.14	0.08	0.494
Velocity RMS app	0.14	0.10	0.17	0.13	0.12	0.06	0.776
Velocity diff RMS	0.01	0.03	0.06	0.07	0.03	0.10	0.082
							
**Strain rate (%/s)**							
Strain rate Mean bpp	0.22	0.09	0.23	0.09	0.21	0.08	0.865
Strain rate Mean app	0.27	0.13	0.31	0.14	0.23	0.10	0.186
Strain rate diff mean	0.05	0.04	0.08	0.05	0.02	0.19	0.150
							
Strain rate RMS bpp	0.28	0.10	0.29	0.11	0.28	0.10	0.910
Strain rate RMS app	0.32	0.16	0.38	0.18	0.27	0.12	0.167
Strain rate diff RMS	0.04	0.06	0.09	0.07	0.01	0.02	0.106
							
**Strain (% compared with rest)**							
Strain Max bpp	64.10	22.51	62.35	22.83	65.59	23.06	0.691
Strain Max app	55.87	23.68	60.25	25.72	52.16	22.16	0.424
Strain diff max	8.24	1.17	2.11	2.89	13.42	0.90	0.424
							
Strain Mean bpp	33.08	11.97	32.32	10.15	33.72	13.71	0.608
Strain Mean app	30.95	13.42	33.68	14.66	28.63	12.38	0.459
Strain diff mean	2.13	1.45	1.36	4.51	5.09	1.32	0.331
							
Strain RMS bpp	39.37	12.46	39.20	12.57	39.52	12.88	0.820
Strain RMS app	34.26	17.03	39.41	17.39	29.90	16.10	0.167
Strain diff RMS	5.11	4.58	0.21	4.82	9.62	3.22	0.150

Comparisons are made between the Trapezius myalgia (TM) and a healthy control group without pain with respect to pain/exercise provocation. The following acronyms are used: before pain provocation (bpp), after pain provocation (app), Max = maximum value, RMS = Root mean square value, Diff = Difference between the value before, and after pain provocation (result is given in absolute value). The results of the statistical analyses between the two groups are given at the far right. The result shows no significant differences on groups level

### Univariate analyses of ultrasound variables – within groupcomparisons

We also made within group analyses with respect to before (i.e., bpp) versus after (i.e., app) the pain/exercise provocation tests (Table 2). No significant differences were found on a group level when all subjects were taken together. Significant differences were found regarding mean strain rate and RMS strain rate in the healthy controls indicating a higher strain rate after exercise than before. There was also a tendency according to the strain RMS parameter (p = 0.055) that indicated that TM patients had a lower activity after pain provocation compared to before.

**Table 2:** Wilcoxon's test. Group differences before and after pain/exercise provocation.

**Variables**	**All** **(p-values)**	**Controls** **(p-values)**	**TM** **(p-values)**
Velocity diff Mean	0.932	0.594	0.463
Velocity diff RMS	0.954	0.328	0.249
Strain rate diff Mean	0.067	0.033	0.701
Strain rate diff RMS	0.170	0.033	0.753
Strain diff Max	0.179	0.859	0.087
Strain diff Mean	0.530	0.929	0.345
Strain diff RMS	0.170	0.929	0.055

No significant differences were found on a group level when all subjects were taken together. Significant differences were found regarding mean strain rate and RMS strain rate in the healthy controls indicating a higher strain rate after exercise than before. There was also a tendency according to the strain RMS parameter (p = 0.055) that indicated that TM patients had a lower activity after pain provocation compared to before. For explanations of abbreviations, see the legend for Table 1.

### The multivariate analysis

It is reasonable to assume that several of the variables shown in Table 1 in fact were closely inter-correlated and differences might exist between the two groups of subjects or within a group. Hence, in the next step of the analyses we made a principal component analysis (PCA). The purpose of this approach was to investigate variable patterns rather than looking for correlations between single variables in isolation. In the multivariate analysis, the velocity variable was removed due to the fact that the velocity parameter measures the global velocity towards and away from the probe and we aimed to focus on muscle specific regional intra-muscular deformation and deformation rates.

**Figure 4:**
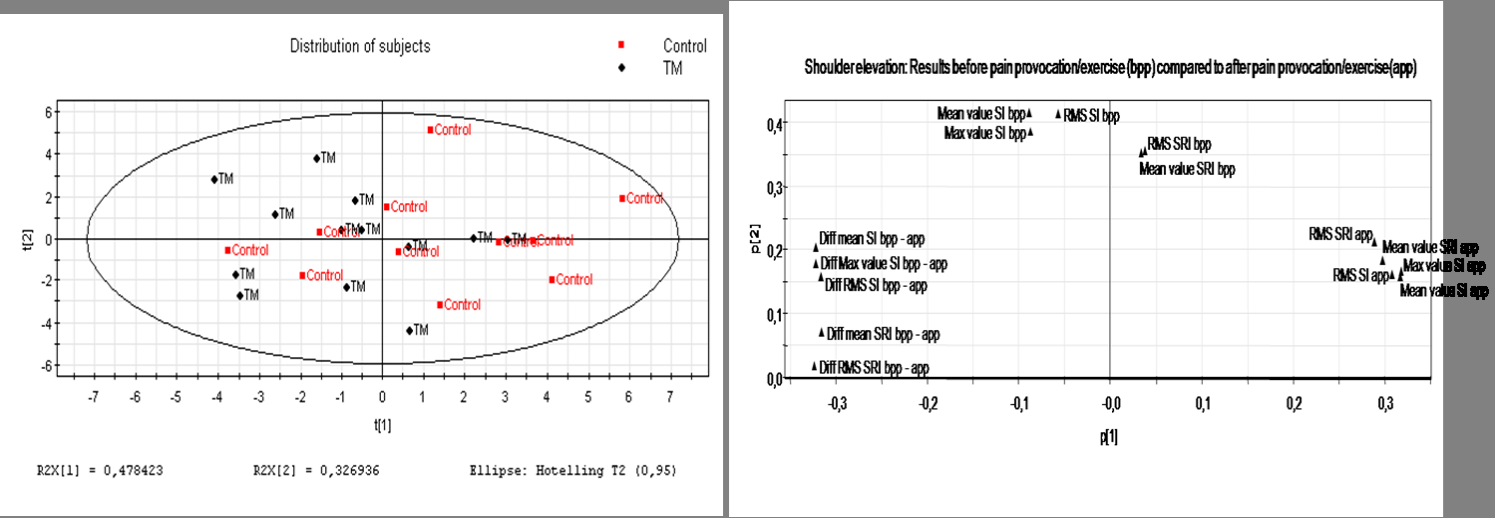
**a: Score plot according to subject distribution**. The score plot depicts the multivariate relationships between the different subjects. The controls are coloured red and the TM patients black. The corresponding plot depicting the relationships between variables (i.e., loading plot) is shown in Figure 4b. **4b: Loading plot according to variable distribution**. Loading plot depicts the multivariate relationships between the variables. For explanations of abbreviations see Table 1. The corresponding plot depicting the relationships between subjects (i.e., score plot) is shown in Figure 4a. The following acronyms are used: before pain provocation (bpp), after pain provocation (app), Max = maximum value, RMS = Root mean square value, Diff = Difference between the value before and after pain provocation, SI = strain (deformation), and SRI = strain rate (deformation rate).

The model calculation generated three significant components explaining 90% of the variance in X (R^2^cumulative: 0.90; R^2^first component: 0.48, R^2 ^second component: 0.33; and R^2 ^third component: 0.09). As the third component only explained 9% of the variation, the graphic overview of the relationships between the subjects and variables are presented according to the first and second components in Figures 4a and 4b. In Figure 4a, the distribution of subjects is depicted according to the projection plane created by the first two latent variables t[1] and t[2] that together explain the largest part of the variance (all together 81% of the variation). A tendency can be seen that most subjects fulfilling the criteria of TM are located in the left part of the plot while most of the healthy controls are located in the right hand part (Figure 4a). However, there is a certain blend between the two groups.

The distribution of variables is presented in Figure 4b. It reveals that all variables describing the before pain situation are well correlated and located in the upper part of the plot and mainly loading on the second component (the vertical axis). The variables responsible for the situation after pain provocation are also well clustered and load mainly positively on the first component. In the same way, the variables explaining the difference between before and after pain provocation are mainly negatively loaded on the first component. These latter clusters of variables are negatively correlated due to the fact that they load with different signs on the first component. The loading plot thus shows that all our variables in fact reflect three variable clusters or new multivariate variables: 1) the before pain provocation variables; 2) the after pain provocation variables; and 3) the variables describing differences between before and after pain provocation.

Comparing the subject plot (Figure 4a) and the variable plot (Figure 4b), the score plot reveals that the control and TM-dominating groups are separated according to the first component whereas the second component is mainly responsible for the intra-group variation.

The loading plot, corresponding to the first principal component, reveals that the separation between the groups is primarily explained by the after pain provocation situation as well as the difference between the variables before and after pain provocation. Hence, this pattern indicates that some variables are more related to the groups of subject in the left part of the graph compared to the right part. The result from the comparison between the plots also reveals that according to the first component the control-dominating group has higher values in all variables after pain provocation while the TM-dominating group in general has lower values. The second component in comparison describes the intra-group variation within the TM and control-dominating group. Therefore, a more fine-tuned analysis is performed below.

### Characteristics and differences between subject groups

**Figure 5:**
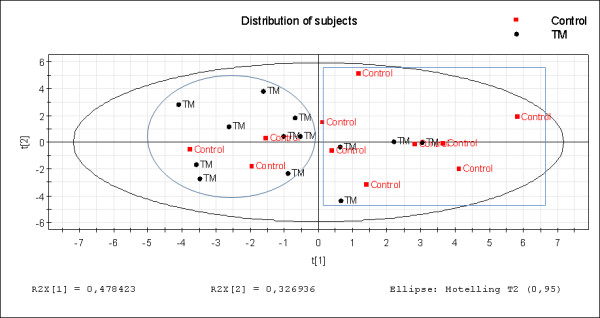
**Clustering the subjects according to distribution in the score plot**. Subjects have been clustered according to the projection onto the score plot to describe what variable profile characterizing each cluster and hence the separating variables. The controls are coloured red and the TM patients black. The ellipsis captures the TM-dominating group while the rectangle captures the Healthy control-dominating group.

To further investigate the characteristics and differences in the left and right part of the plot in Figure 4a, the subjects were divided into two groups in respect to its location within the observation plot (figure 5).

**Figure 6:**
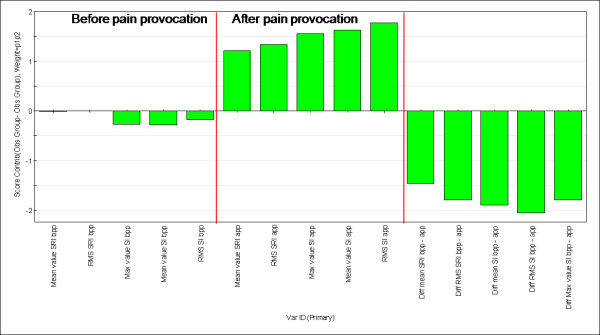
**The variable contribution profile explaining the separation between the groups (ellipsis versus rectangle) in Figure 5**. The variable profile plot describes in detail the contribution of each variable with respect to values before and compared to after pain provocation. Furthermore, the last five variables represent the difference value. Hence, before pain provocation, i.e., the first five piles, no difference is seen comparing the values before and after pain provocation. The piles 6 to 10 all show lower values according to the TM group (in the region of 2 SD). The last five piles confirm the difference before compared to after pain provocation.

The result of the comparison between the subjects captured by the ellipsis and the rectangle respectively is presented as a resulting variable contribution profile (Figure 6). The distribution of TM and controls differed significantly between the ellipsis and the rectangle (p = 0.050, one-tailed).

The results confirm the indication above: there is no major difference between the two groups before pain provocation, but the TM-dominating groups have lower levels concerning the variables after pain provocation compared with the control-dominating group. Furthermore, there is a larger difference between the values before and after pain provocation in the TM-dominating group. The result of the analyses shown in Figures 5c describes how the variables have changed – measured before and after pain provocation – when comparing the TM and non-pain groups.

## Discussion

The following important results and conclusions of the present pilot study will be discussed below:

• Tissue velocity imaging, as a method, seems sensitive to alterations in muscle tissue response and therefore might effectively describe musculoskeletal tissue activity and dynamics.

• It was possible to identify *clusters of subjects *who share similar tissue responses according to tissue velocity measurements during a standardized shoulder elevation.

• It was possible to describe group specific *variable profiles *that indicate differences among the subjects and these differences seem to be related to whether the subject had chronic pain or was a healthy control.

### Methodological considerations

Tissue velocity imaging measures velocities in the axial direction, i.e., in the direction towards and away from the probe. This is a drawback as the fascicles of the upper trapezius are distributed in a more horizontal direction. However, the direction of fascicles according to the ultrasound images is relatively homogeneous. Based on the incompressibility of muscles (i.e., if a muscle is compressed in one direction it has to expand in the other directions) an indirect measurement is still possible. It is also possible to confirm the contraction by visual inspection of the ultrasound loops where the diameter of the muscle belly becomes thicker/thinner as the shoulder elevation enters either of the concentric or eccentric phases of the movement. During elevation, the muscle expands in a vertical direction. Therefore, we used relative rather than absolute measurements.

Another methodological aspect concerns the fact that the probe is handheld. Of course reproducibility can be questioned. However, reproducibility according to cardiac applications that are similar to the situation of registering motions from a skeletal muscle has been shown to be reliable [41,42]. To optimize the location of the probe, the SENIAM standard point was used for centering the probe and the contour of the probe was drawn. Yet a moment of uncertainty occurs when the angle of the probe is placed on the trapezius muscle, but as far as possible the applicator is aware of this fact and was as careful as possible to achieve the same projection image.

As this is a pilot study, further studies are needed in order to confirm the results indicated in this study. To improve the study design, full EMG should be provided. Interpretations can then be with respect to the relationship between neuromotor activation and muscle tissue response. This matter will be presented in a forthcoming study. Pain intensity ratings should also be made after the first two elevations to evaluate the intermediate pain ratings. Also a larger study including more patients would be of great value.

The number of performed repetitions could have been chosen differently. There is a variation in pain intensity between 6–8 (VAS) in the patient group. It is in accordance with a reasonable variation as the response to a certain activity is not easy to foresee and the estimation according to the VAS scale is not a direct correlation to biological activity. The VAS scale is used as a reference instrument to standardize a cut off for the purpose of providing a moderate pain rather than a mild pain.

It could be argued that some subjects used less force after pain provocation and thus lower degree of activation and this can of course be a relevant objection. In this study, the task to be performed was not a heavy one. On the contrary, a 3-cm shoulder elevation carrying a weight of just 1 kg was performed. The shoulder elevation was short and standardized in terms of height of elevation and tempo of the movement. The interesting finding from a qualitative point of view is that less of the muscle seems to be contracted and this part is in the middle/deeper part surrounded by more passive trapezius muscle tissue segments after pain provocation compared to before pain provocation

In future studies it will be an advantage if the applicator of the US registration is blinded with respect to group belonging.

Multivariate statistics have been used in this pilot study. The strength of this procedure is that during the analysis subjects are not clustered a priori, e.g., in categories as patients and controls. Instead, subjects are seen as uncategorized and are graphically projected based on the results from the variable analysis. Furthermore, the variables have been scaled and mean-centred so each variable has the same impact, i.e., the same possibility to affect the model. Yet another benefit is that clusters of subjects can be related to a *variable profile *rather than just a single variable: the distribution of variables provides patterns of variables that in turn describe the internal variable structure in relation to the identified cluster of subjects.

### Before pain provocation/exercise

This study focuses on the intra-muscular activity in the upper part of the trapezius muscle. Strain rate is a variable that represents the rate at which small (8 mm) muscle tissue segments deform and hence contribute as an important variable to describe muscle activity and intra-segmental coordination pattern. Because the rate of deformation is calculated by the tissue velocity algorithm, the presence of force is at least to some extent part of the measurement. Hence, strain rate can also be thought of in terms of contractility. Strain, on the other hand, is a regional tissue deformation parameter that presents muscle tissue activity as an additive presentation of a regional tissue progress as a function of the progress of a movement. In other words, there are no rapid fluctuations between positive and negative strains as a consequence of muscle activation as is the case in the strain rate parameter, but instead the gradual distribution of tissue contraction beyond a certain degree of deformation is compared to rest. The benefit of the strain parameter is that a certain degree of deformation can be chosen and hence variations in the coordination pattern can be visualised and compared as a function of a certain degree of deformation. As a consequence, activation patterns according to fine-tuned tissue activations can also be studied.

It is interesting that strain rate and strain did not differ significantly before pain provocation, neither according to the univariate (Table 1) or multivariate analyses (Figures 4a and 4b). This could be seen as indicating a relative homogeneous reference movement. One objection to this finding could be that the TM group is weaker than the controls [43]. In the present study, the strength was not measured. Clearly, the importance of this factor cannot be determined. Hence, future studies ought to consider aspects of biomechanical output, such as strength and fatigue, when validating the tissue velocity method within this application.

### After pain provocation/exercise

The univariate analysis revealed significant differences according to mean strain rate and RMS strain rate in the healthy controls indicating a higher strain rate after exercise than before. A combination of a warming-up effect together with a dynamic muscle tissue could be one explanation of the increase of the deformation rate after several repetitions. The multivariate analyses (Figures 4a,b, 5 and 6) revealed that patients with chronic trapezius myalgia after pain provocation due to exercise on a group level showed decreased strain and unchanged strain rate whereas healthy controls had unchanged strain and increased strain rate.

In healthy subjects, from a muscle physiological point of view, these results taken together could be interpreted as a more dynamic tissue. Possible explanations for these findings might be warming-up effects due to increased blood flow (exercise hyperemia) and increased neuromuscular control. The pain in itself may also be factor to be considered. Studies have shown that the interplay and coordination according activities in prime and secondary movers may be affected by pain in the sense that a decrease in an agonist muscle activity may result in an increase in an antagonist muscle activity [13-17,19]. In future studies of healthy subjects, it is important to analyze whether other factors – such as sex, degree of fitness, strength, and fatigue – influence strain and strain rate both in prime movers and postural muscles

In agreement with the physiological interpretation above, the TM group has a less flexible or dynamic muscle tissue as a consequence of exercise and pain provocation. As briefly referred in the introduction, several studies indicate morphological, biochemical, and neuromuscular alterations in chronic trapezius myalgia when compared to healthy controls. The morphological alterations, such as increased prevalence of RR-fibres, are less likely as direct explanations to decrease strain. If such changes were important for strain and strain rate, then a lower strain would reasonably have been observed before pain provocation. This was not the case, however.

Based on studies using microdialysis of the trapezius, our group has reported that patients with chronic work-related trapezius myalgia have increased interstitial concentrations of potassium, serotonin, lactate, pyruvate, and glutamate at rest [9,10]. The interstitial concentrations of these substances also increase during brief exercise associated with a significant increase in pain intensity. One possible explanation could be that these concentrations are so high, even though exercise hyperaemia, that they directly affect the mechanical properties of the active muscle fibres.

A third explanation is related to altered neuromuscular control due to increased pain as a consequence of exercise. Le Pera et al. [44] describe how motor control mechanisms are affected by muscle pain by way of reflex and central mechanisms. Experimental studies modulating the noxious system also demonstrate altered responses to acute pain due to injections of algogenic substances, mechanical pressure, ischemia, and electrical stimulation [45,46]. Injecting hypertonic saline into the upper trapezius muscle resulted in a decreased EMG activity both during static [46,47] and dynamic tasks [48]. Acute pain can result in reorganization among trapezius muscle subdivisions during repetitive shoulder flexions [49]. Changes in the pattern of coordination during parts of the contraction cycle have been shown during dynamic activities in patients with chronic pain [13-16]. Although differences have been observed at a group level, it is obvious that not all patients have altered coordination patterns in the trapezius muscle [50], which is interesting with respect to the fact that we found a certain blend between controls and TM. The studies are in reasonable agreement with the pain-adaptation model [51,52]. This model states that a decrease in agonist muscle activity and increase in antagonist muscle activity is a consequence of pain. Hence the decreased strain and unchanged strain rate in TM could be a consequence of such an altered neuromuscular control resulting in a less activated trapezius as a consequence of the increasing pain due to exercise. Another contributing factor in this respect could be a hyper-sensitised pain system (i.e., central sensitization and/or decreased descending control) as a consequence of plastic changes in the pain system due to nociceptive impulses of long duration in TM.

## Conclusion

This pilot study indicates that tissue velocity imaging, as a method, seems promising as a way to describe musculoskeletal tissue activity and dynamics in patients with chronic pain conditions. The study also indicates an altered muscle tissue dynamic after pain provocation/exercise among the majority of trapezius myalgia patients compared with the healthy controls. In future studies that attempt to validate the tissue velocity method, it is desirable to validate the technique in other movements and against biomechanical output (e.g., strength and fatigue), multichannel surface-EMG, interstitial situation (i.e., using microdialysis), or muscle morphological characteristics

## Abbreviations

App: After pain provocation; Bpp: Before pain provocation; Diff: Difference; dL: The derivative according to length; DModX: Distance to model among the X-variables; dt: The derivative according to time; L: The measured new length of the muscle after activation; L_0_: The original length before muscle activation; Q^2^: The degree of prediction of a model; R^2^: The degree of explained variation of a model; RMS: Root mean square; SD: standard deviation; SI: Strain imaging; SRI: Strain rate imaging; TM: Trapezius myalgia; TVI: Tissue velocity imaging.

## Competing interests

The authors declare that they have no competing interests.

## Authors' contributions

MP: Study design, performer of the study, analyzing and interpreting data, author of manuscript. BL: Selecting and including patients based on journals, critically revising manuscript. LÅB: Co-developer of the analyzing software package, critically revising manuscript. BG: Study design, interpreting data, author of manuscript.

## Pre-publication history

The pre-publication history for this paper can be accessed here:


